# Zearalenone Induces Endothelial Cell Apoptosis through Activation of a Cytosolic Ca^2+^/ERK1/2/p53/Caspase 3 Signaling Pathway

**DOI:** 10.3390/toxins13030187

**Published:** 2021-03-04

**Authors:** Hyeon-Ju Lee, Se-Young Oh, Inho Jo

**Affiliations:** Graduate Program in System Health Science and Engineering, Department of Molecular Medicine, College of Medicine, Ewha Womans University, 25 Magokdong-ro-2-gil, Gangseo-gu, Seoul 07804, Korea; eihj0323@hanmail.net (H.-J.L.); ohs@ewha.ac.kr (S.-Y.O.)

**Keywords:** mycotoxin, zearalenone, apoptosis, endothelial cells, calcium

## Abstract

Zearalenone (ZEN) is a mycotoxin that has been reported to damage various types of cells/tissues, yet its effects on endothelial cells (ECs) have never been investigated. Therefore, this study investigates the potential effects of ZEN using bovine aortic ECs (BAECs). In this study, we found that ZEN induced apoptosis of BAECs through increased cleavage of caspase 3 and poly ADP-ribose polymerase (PARP). ZEN also increased phosphorylation of ERK1/2 and p53, and treatment with the ERK1/2 or p53 inhibitor reversed ZEN-induced EC apoptosis. Transfection of BAECs with small interfering RNA against ERK1/2 or p53 revealed ERK1/2 as an upstream target of p53 in ZEN-stimulated apoptosis. ZEN increased the production of reactive oxygen species (ROS), yet treatment with the antioxidant did not prevent EC apoptosis. Similarly, blocking of estrogen receptors by specific inhibitors also did not prevent ZEN-induced apoptosis. Finally, chelation of cytosolic calcium (Ca^2+^) using BAPTA-AM or inhibition of endoplasmic reticulum (ER) Ca^2+^ channel using 2-APB reversed ZEN-induced EC apoptosis, but not by inhibiting ER stress using 4-PBA. Together, our findings demonstrate that ZEN induces EC apoptosis through an ERK1/2/p53/caspase 3 signaling pathway activated by Ca^2+^ release from the ER, and this pathway is independent of ROS production and estrogen receptor activation.

## 1. Introduction

Zearalenone (ZEN) is a common mycotoxin produced by the *Fusarium* species, and is a frequent contaminant of crops, grains and food products [1]. ZEN can be converted into reduced metabolites, such as α-zearalenol, β-zearalenol, α-zearalanol, β-zearalanol and zearalanone [2]. ZEN and some of its metabolites are known to exhibit estrogenic activity and disrupt endocrine functions in animals [3,4]. The molecular mode of action of ZEN in mimicking estrogen activity has been established by many studies. It has been proposed that ZEN binds competitively to genomic or nongenomic estrogen receptors to activate transcription of estrogen-responsive genes, eventually leading to estrogen/estrogen receptor-mediated cellular effects [5,6]. These actions may consequently disrupt reproductive functions in variety of cells and animals; for example, intake of ZEN for 70 days decreased sperm concentration and altered the morphology of spermatozoa in male mice [7].

Apart from the estrogenic activity of ZEN, ZEN also mediates other toxicological effects through various mechanisms [8,9]. For example, ZEN increases intracellular reactive oxygen species (ROS) production, in various cell types, including porcine small intestinal epithelial cell line SIEC02, mouse Sertoli TM4 cells [10] and human neuroblastoma cell line SHSY-5Y cells [11]. ZEN-induced ROS production also mediates endoplasmic reticulum (ER) stress to reduce proliferation and increase apoptosis of mouse Leydig cells exposed to ZEN in vitro [12]. Another study also showed that ZEN exposure induces pro-apoptotic protein Bax, promoting cytochrome c release from mitochondria into the cytosol, which activates caspase 9 and 3 followed by cleavage of poly ADP-ribose polymerase (PARP) in Leydig cells [13]. Other apoptotic transcription factors, such as the c-jun NH_2_-terminal kinase (JNK) and p38 mitogen-activated protein kinase (MAPK) (p38 MAPK), were also shown to be induced in murine macrophage cell line RAW264.7 exposed to ZEN [14]. These studies clearly suggest that ZEN activates various molecular pathways to induce cell apoptosis, thereby damaging its target cells/tissues.

Many studies have used endocrine and reproductive cells to assess the toxicological effects of ZEN, but the potential effects on endothelial cells (ECs) have been neglected, despite their potential biological relevance. ECs are one of essential components of the vascular system. Once drugs and/or chemicals are ingested, they first contact the EC lining of the blood vessels and other cellular matrices before reaching their target sites. Studies have shown that ECs can be easily damaged by various stressors, such as cigarette aerosol, H_2_O_2_, and oxygen-glucose deprivation/reperfusion [15,16,17]. These factors can cause DNA damage and inflammation, which eventually lead to death of ECs, and weaken the integrity and function of blood vessels.

ZEN is often found in animal feed, altering the reproductive function of animals. Previous studies showed that ZEN changes the serum level of progesterone and estradiol and induces teratogenic effects in pigs and sheep [18]. Bovine species are also considered as a major exposure group to mycotoxins including ZEN, and bovine aortic ECs (BAECs) have been suggested as a good model for functional studies on ECs due to their ease of using molecular techniques such as gene transfection and modification. For these reasons, we investigated the potential effects of ZEN using BAECs.

## 2. Results

### 2.1. Zearalenone Increases Apoptotic Death of BAECs

**Figure 1:**
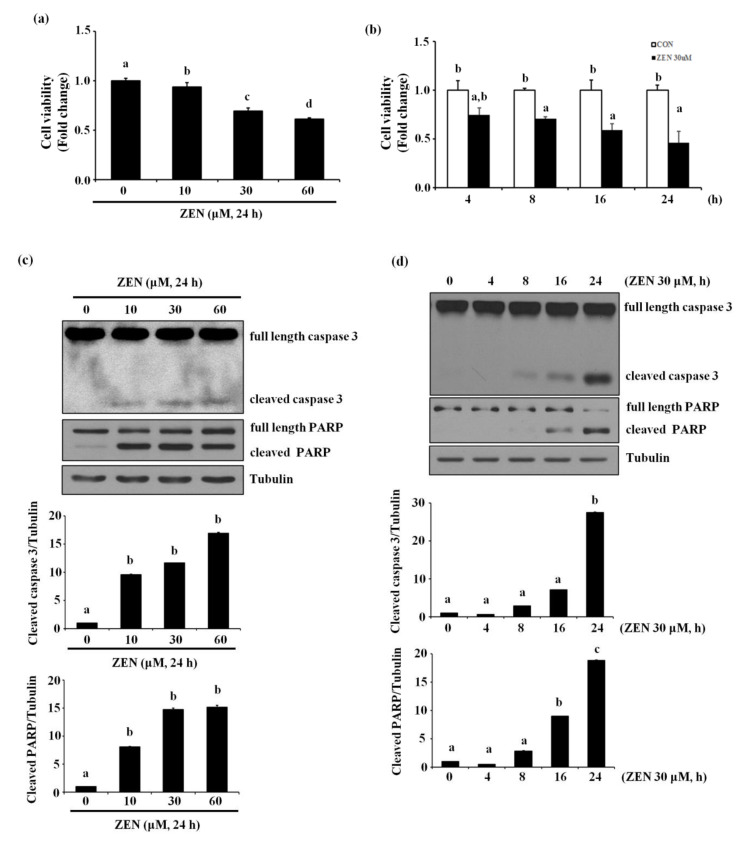

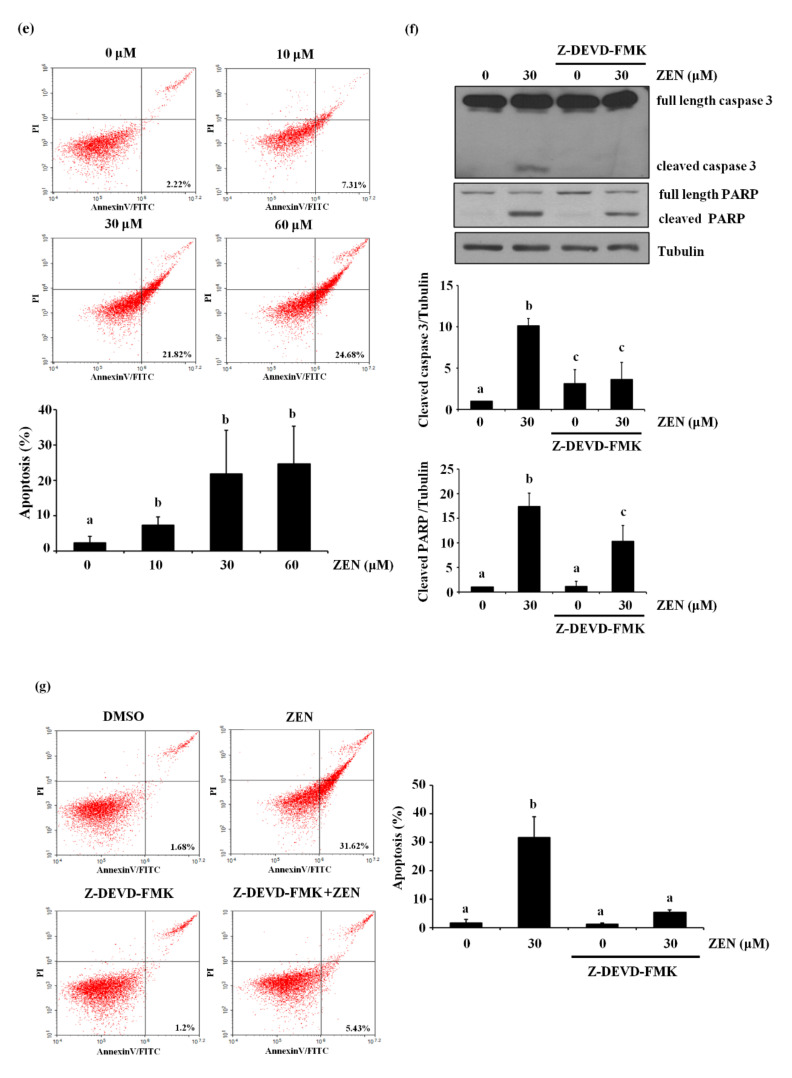
ZEN reduces the viability of BAECs by caspase-dependent apoptosis. BAECs were treated with various concentrations of ZEN (0, 10, 30 or 60 µM) for 24 h or 30 µM of ZEN for various time points (0, 4, 8, 16 or 24 h). (**a**,**b**) Cell viability was measured using the MTT assay. (**c**,**d**) The protein expression of cleaved caspase 3 and PARP in the BAECs was quantified (relative to relative to tubulin) using western blot analyses. (**e**) Apoptosis induced by ZEN at different concentrations was measured by FACS using annexin V/PI staining. After the pretreatment of 20 µM Z-DEVD-FMK for 1 h, BAECs were incubated with 30 µM ZEN for 24 h. (**f**) The protein expression of cleaved caspase 3 and PARP relative to tubulin was quantified using western blot analyses. (**g**) Apoptosis was measured by FACS using annexin V/PI staining. The plots depict the mean fold changes relative to the control (±SD) from at least four independent experimental trials. The different alphabetical letters refer to significant difference (*p* < 0.05) among groups, which were determined by one-way ANOVA followed by Tukey’s multiple comparisons.

ZEN has diverse effects depending on species, cell types as well as exposure concentrations and time. Concentration ranges of ZEN between 10 pM and 300 μM have generally been used to assess acute toxicity of ZEN in several previous studies [19]. Our study tested various exposure conditions of ZEN on BAECs, and determined that ZEN reduced the viability of BAECs in a concentration- and time-dependent manner (Figure 1a,b). Treatment with 30 μM ZEN for 24 h significantly reduced BAEC viability to ~60% compared with the unexposed control group, and thus, all subsequent experiments were carried out using 30 μM ZEN treatment for 24 h, unless specifically stated otherwise. As shown in Figure 1c,d, ZEN increased cleavage of caspase 3 and PARP in a concentration- and time-dependent manner. An apoptosis assay using annexin V-FITC/PI staining revealed that 2.22, 7.31, 21.82 and 24.68% of BAECs underwent apoptotic cell death when treated with 0, 10, 30 and 60 μM of ZEN, respectively (Figure 1e). When Z-DEVD-FMK, a caspase 3 inhibitor, was co-treated with 30 μM ZEN, cleavage of caspase 3 and PARP was significantly prevented (Figure 1f), and apoptotic cell death induced by ZEN was reduced from 31.62% to 5.43% (Figure 1g). These results indicate that ZEN decreases BAEC viability at least partially through a caspase 3-dependent apoptotic pathway.

### 2.2. ZEN-Induced Apoptosis Is Mediated through Phosphorylation of ERK1/2

**Figure 2:**
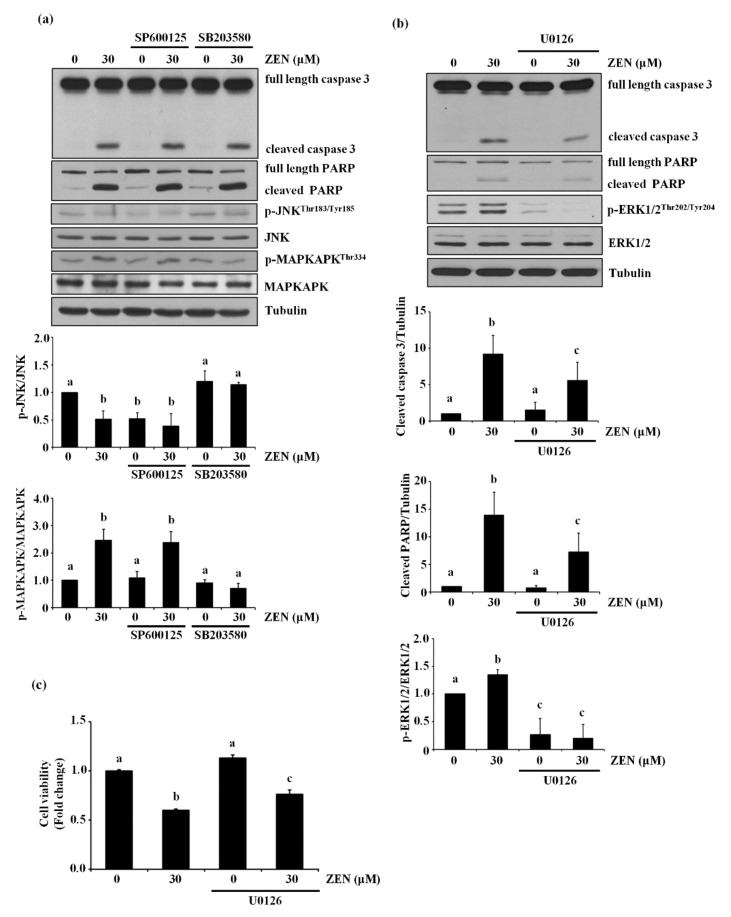
ERK1/2 mediates apoptosis by ZEN in BAECs. (**a**) The protein expression of cleaved caspase 3 and PARP relative to tubulin was quantified using western blot analyses in BAECs exposed to 30 µM of ZEN for 24 h after pretreatment with JNK inhibitor SP600125 (1 μM) or p38 MAPK inhibitor SB203580 (5 μM) for 1 h. SB203580 does not directly affect phosphorylation of p38, but inhibits p38 catalytic activity by binding to the ATP binding pocket, inhibiting phosphorylation of MAPKAPK, a downstream molecule of p38 MAPK. The plots are representative of at least four independent experimental trials. (**b**) After pretreatment with 1 µM of ERK1/2 inhibitor U0126 for 1 h, BAECs were incubated with 30 µM of ZEN for 24 h. The protein expression of cleaved caspase 3 and PARP, p-ERK1/2, and ERK1/2 relative to tubulin was quantified using western blot analyses. (**c**) Cell viability was measured using the MTT assay. The plots depict the mean fold changes relative to the control (±SD) from at least four independent experimental trials. The different alphabetical letters refer to significant difference (*p* < 0.05) among groups, which were determined by one-way ANOVA followed by Tukey’s multiple comparisons.

The MAPK proteins JNK, p38 MAPK and ERK1/2 have been reported to be common apoptotic signaling molecules [20,21]. In particular, JNK and p38 MAPK were reported to mediate ZEN-induced apoptosis in RAW264.7 cells [14]. Based on these previous findings, we examined the role of JNK and p38 MAPK in ZEN-induced apoptosis of the BAECs. However, neither treatment with SP600125, a JNK inhibitor, nor SB203580, a p38 MAPK inhibitor, inhibited the effect of ZEN on cleavage of caspase 3 and PARP, suggesting no involvement of these two kinases in the ZEN-induced apoptosis (Figure 2a). When ERK1/2, another protein of the MAPK family, was selectively inhibited using U0126, the expression of cleaved caspase 3 and PARP induced by ZEN exposure was reduced (Figure 2b). As expected, treatment with U0126 also prevented the apoptosis induced by ZEN treatment (Figure 2c). Together, these data suggested that ERK1/2 plays an important role in enhancing ZEN-induced apoptosis of BAECs.

### 2.3. p53 Is Involved in ZEN-Induced Apoptosis of BAECs

**Figure 3:**
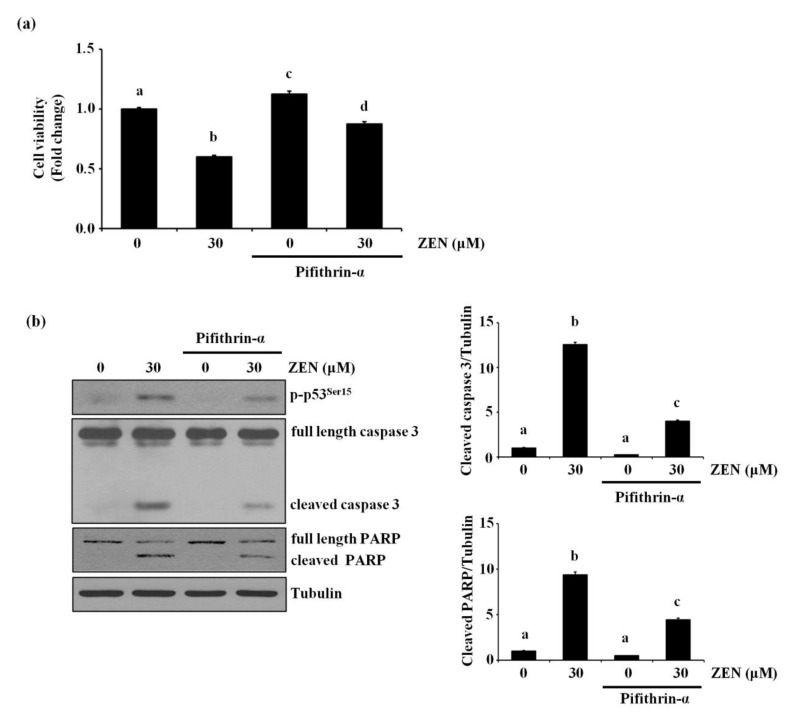
p53 is involved in EC apoptosis by ZEN. After pretreatment with 5 µM of p53 inhibitor pifithrin-α for 1 h, BAECs were incubated with 30 µM of ZEN for 24 h. (**a**) Cell viability was measured by using the MTT assay. (**b**) The protein expression of cleaved caspase 3 and PARP, and p-p53 relative to tubulin was quantified using western blot analyses. The plots depict the mean fold changes relative to the control (±SD) from at least four independent experimental trials. The different alphabetical letters refer to significant difference (*p* < 0.05) among groups, which were determined by one-way ANOVA followed by Tukey’s multiple comparisons.

Since p53 is reported to mediate ZEN-induced apoptosis in HepG2 and RAW264.7 cells [14,22], we examined whether p53 was also involved in ZEN-induced EC apoptosis under our experimental conditions. Inhibition of p53 using pifithrin-α reversed the apoptosis induced by ZEN as shown in Figure 3a. As expected, we also found that cleavage of caspase 3 and PARP was significantly reduced in cells treated with the p53 inhibitor (Figure 3b). These results indicated that p53 was involved in ZEN-induced apoptosis of BAECs.

### 2.4. ERK1/2 Is an Upstream Mediator of p53-Mediating ZEN-Induced EC Apoptosis

**Figure 4:**
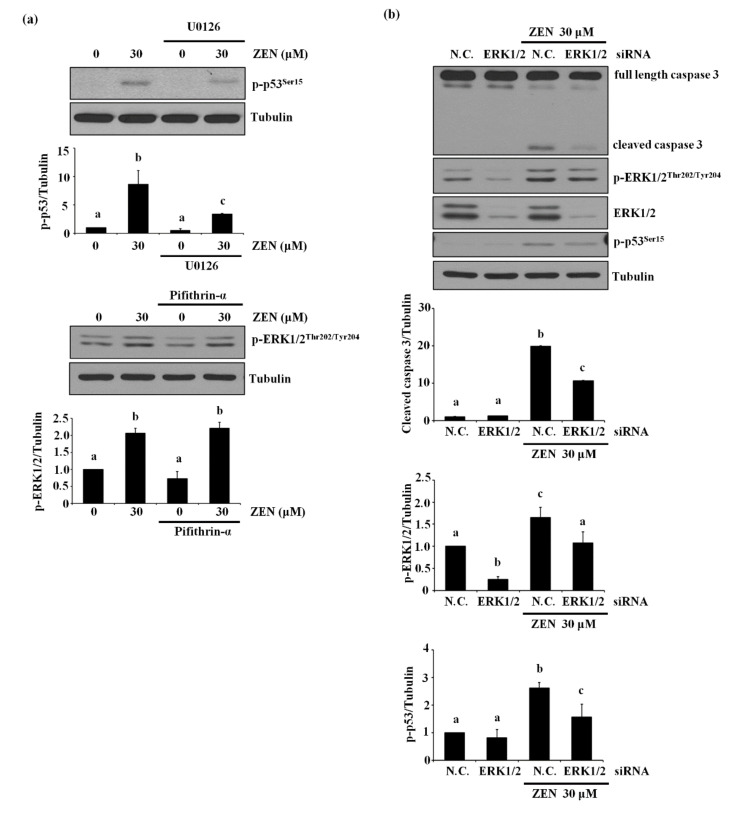

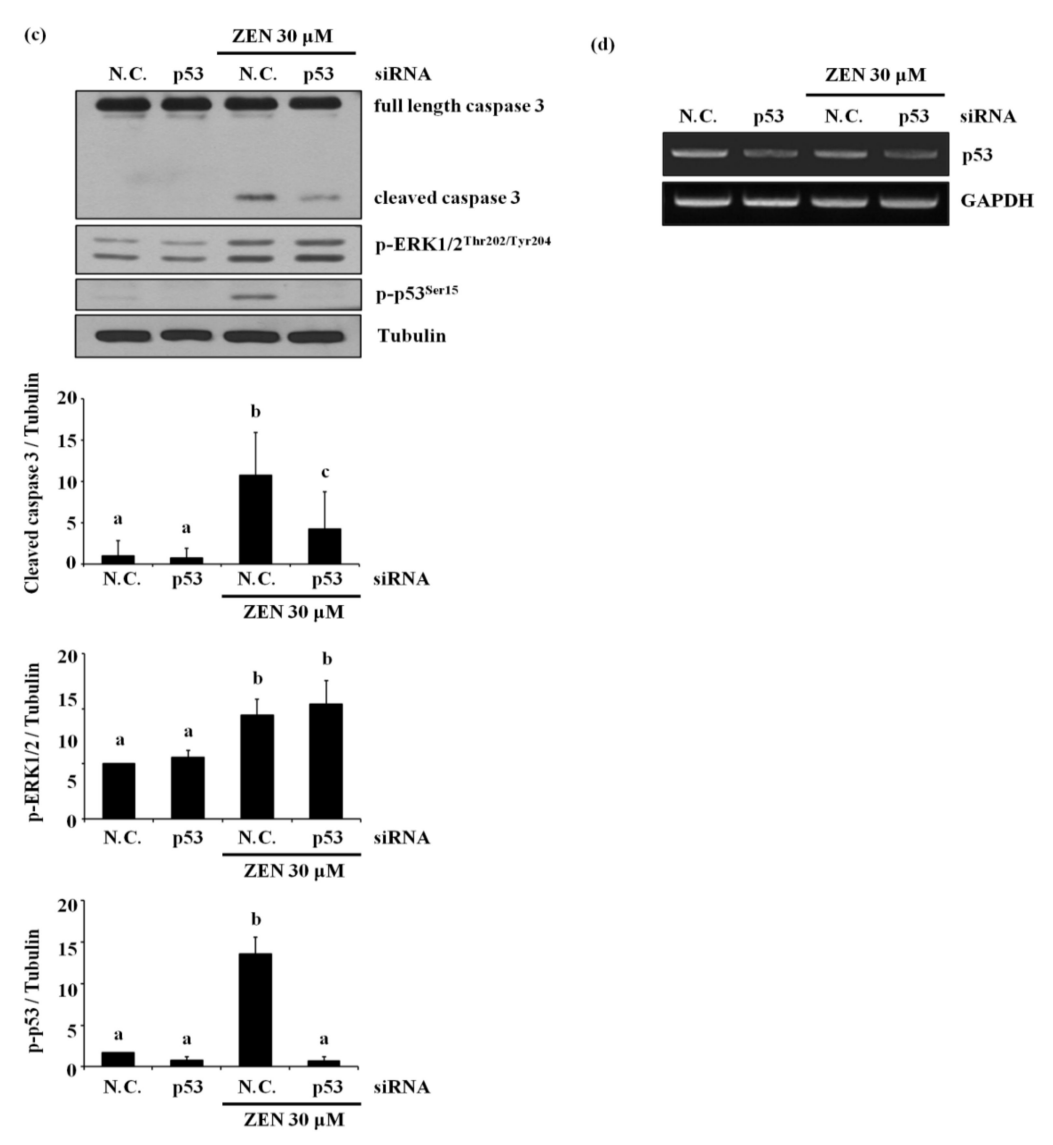
ZEN-induced EC apoptosis is mediated via a signaling axis of ERK1/2/p53/caspase 3. (**a**) After pretreatment with 1 µM of U0126 or 5 µM of pifithrin-α for 1 h, BAECs were incubated with 30 µM of ZEN for 24 h, and the protein expression of p-p53 and p-ERK1/2 relative to tubulin was quantified using western blot analyses. The plots depict the mean fold changes relative to the control (±SD) from at least four independent experimental trials. (**b**,**c**) The protein expression of cleaved caspase 3 and PARP relative to tubulin were quantified using western blot analyses in the BAECs transfected with siRNA of ERK1/2 and p53 with or without 30 µM of ZEN exposure for 24 h. (**d**) The mRNA expression of p53 relative to GAPDH in the BAECs were quantified using RT-PCR analyses. The plots depict the mean fold changes relative to the control (±SD) from at least four independent experimental trials. The different alphabetical letters refer to significant difference (*p* < 0.05) among groups, which were determined by one-way ANOVA followed by Tukey’s multiple comparisons.

In light of the above results showing the involvement of two proteins, ERK1/2 and p53, in the ZEN-induced apoptosis, we explored which of the two molecules was the upstream mediator of this signaling pathway. As shown in Figure 4a, the inhibition of ERK1/2 using U0126 prevented ZEN-induced phosphorylation of p53. In contrast, inhibition of p53 using pifithrin-α did not alter ZEN-induced phosphorylation of ERK1/2. These results suggest that phosphorylation of p53 is partially dependent on the phosphorylation of ERK1/2 in the ZEN-induced apoptotic pathway. This finding was further validated by the use of siRNA against ERK1/2 or p53 (Figure 4b–d), where siRNA against ERK1/2 reduced ZEN-induced phosphorylation of p53 but not vice versa. Our results indicated that ZEN induces apoptotic death of BAECs by a signaling axis of the ERK1/2/p53/caspase 3.

### 2.5. The Estrogen Receptor Is Not Involved in ZEN-Stimulated Apoptosis

**Figure 5:**
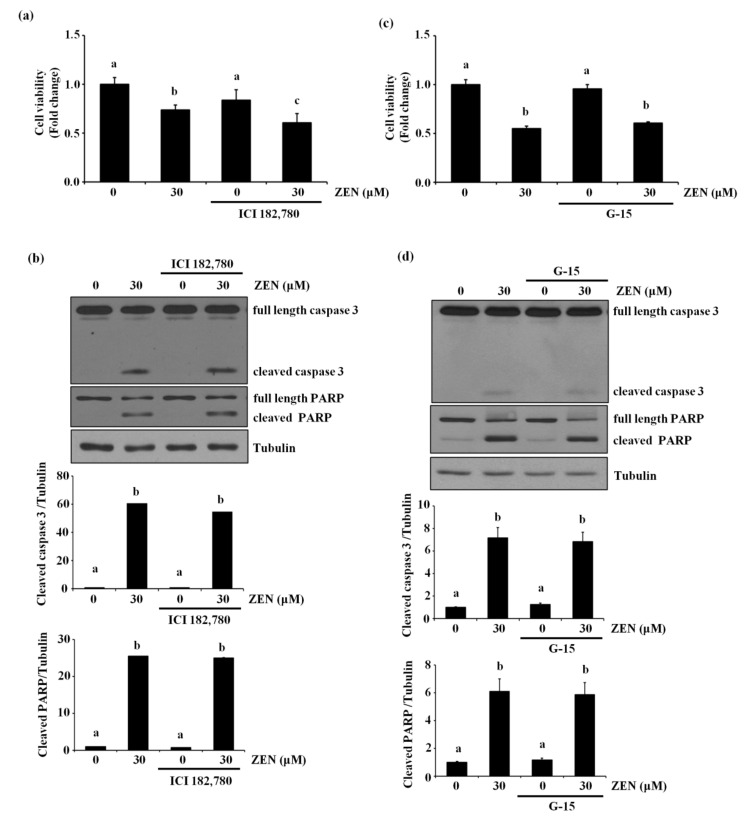
Estrogen receptors do not mediate ECs apoptosis by ZEN. After pretreatment with (**a**,**b**) 10 µM of ICI 182,780 or (**c**,**d**) 1 µM of G-15 for 1 h, BAECs were incubated with 30 µM of ZEN for 24 h. (**a**,**c**) Cell viability was measured using the MTT assay. (**b**,**d**) The protein expression of cleaved caspase 3 and PARP relative to tubulin was quantified using western blot analyses. The plots depict the mean fold changes relative to the control (±SD) from at least four independent experimental trials. The different alphabetical letters refer to significant differences (*p* < 0.05) among groups, which were determined by one-way ANOVA followed by Tukey’s multiple comparisons.

Previously, it has been reported that ZEN exhibits some of its toxic effects through either genomic and/or nongenomic estrogen receptor-mediated signaling pathways [5,6]. Therefore, we examined whether the estrogen receptor was also involved in ZEN-induced apoptotic cell death. Treatment with ICI 182,780, a genomic estrogen receptor antagonist, did not alter cell viability and the levels of cleaved caspase 3 and PARP in BAECs treated with ZEN (Figure 5a,b). Similarly, G-15, a nongenomic estrogen receptor antagonist, also did not prevent ZEN-induced EC apoptosis (Figure 5c,d). Together, these results suggested that ZEN was not likely to induce apoptosis of BAECs through either the genomic or nongenomic estrogen receptor. As shown in Figures S1 and S2, we also confirmed that the concentrations and conditions of the estrogen receptor antagonists used in this experiment were effective enough to negate the estrogen-mediated response by ZEN; ICI 182,780 reversed the endothelial nitric oxide synthase (eNOS) mRNA expression induced by 17β-estradiol, and G-15 decreased the phosphorylation of eNOS at serine 1179 induced by G-1, a nongenomic estrogen receptor agonist, respectively.

### 2.6. ZEN Induces Reactive Oxygen Species (ROS), But ROS Are Not Involved in ZEN-Induced EC Apoptosis

**Figure 6:**
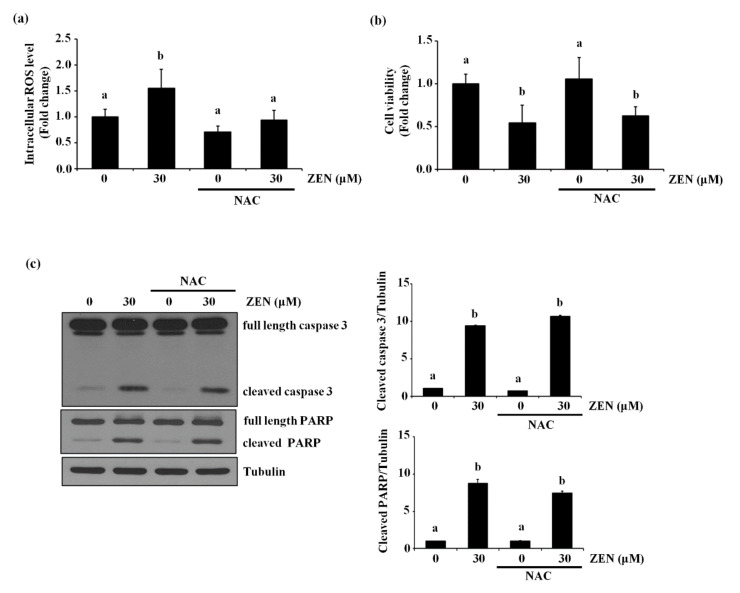
ZEN induces EC apoptosis in an ROS-independent manner. After pretreatment with 5 mM of NAC for 3 h, BAECs were incubated with 30 µM of ZEN for 24 h. (**a**) The intracellular ROS production from the BAECs was measured using DCF-DA. (**b**) Cell viability was measured by using the MTT assay. (**c**) The protein expression of cleaved caspase 3 and PARP relative to tubulin was quantified using western blot analyses. The plots depict the mean fold changes relative to the control (±SD) from at least four independent experimental trials. The different alphabetical letters refer to significant difference (*p* < 0.05) among groups, which were determined by one-way ANOVA followed by Tukey’s multiple comparisons.

Several studies showed that ZEN induces apoptosis by elevating intracellular ROS level [10,11]. To confirm whether ROS also plays a role in ZEN-induced apoptosis of ECs under our conditions, BAECs were treated with NAC, an antioxidant, followed by ZEN exposure. As shown in Figure 6a, ZEN significantly induced ROS production in BAECs, but ROS production induced by ZEN was prevented by NAC treatment. The NAC treatment did not reverse the effects of ZEN on cell viability and apoptosis (Figure 6b,c), indicating that ROS is unlikely to be responsible for the apoptotic death of BAECs induced by ZEN under our experimental conditions.

### 2.7. ZEN Mediates Apoptosis through a Cytosolic Ca^2+^-Dependent Pathway

**Figure 7:**
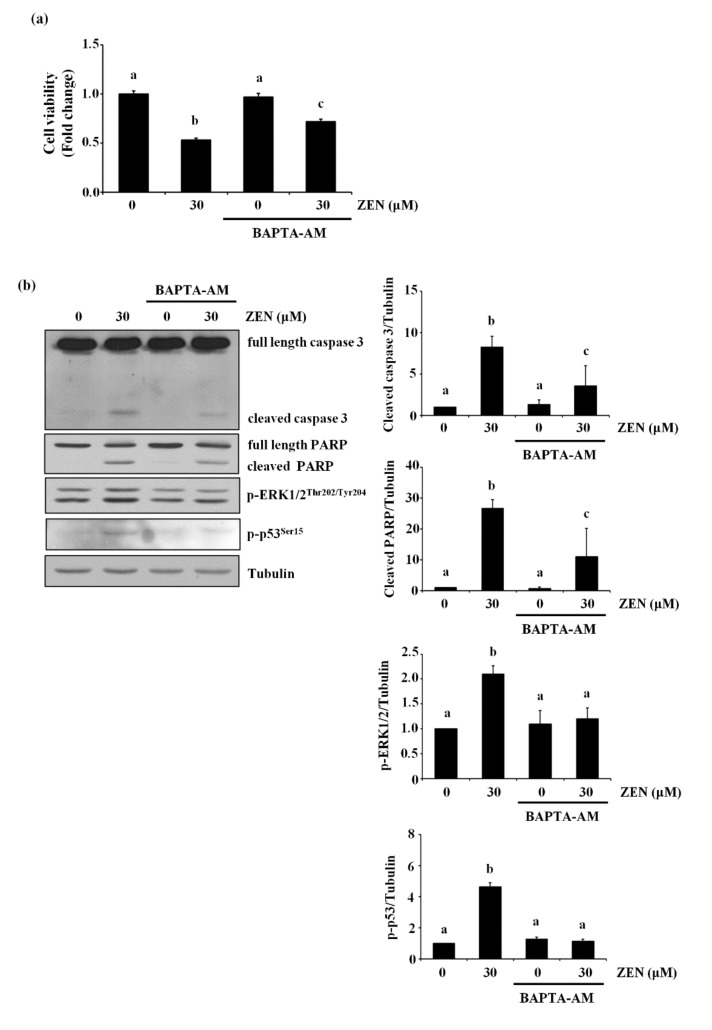

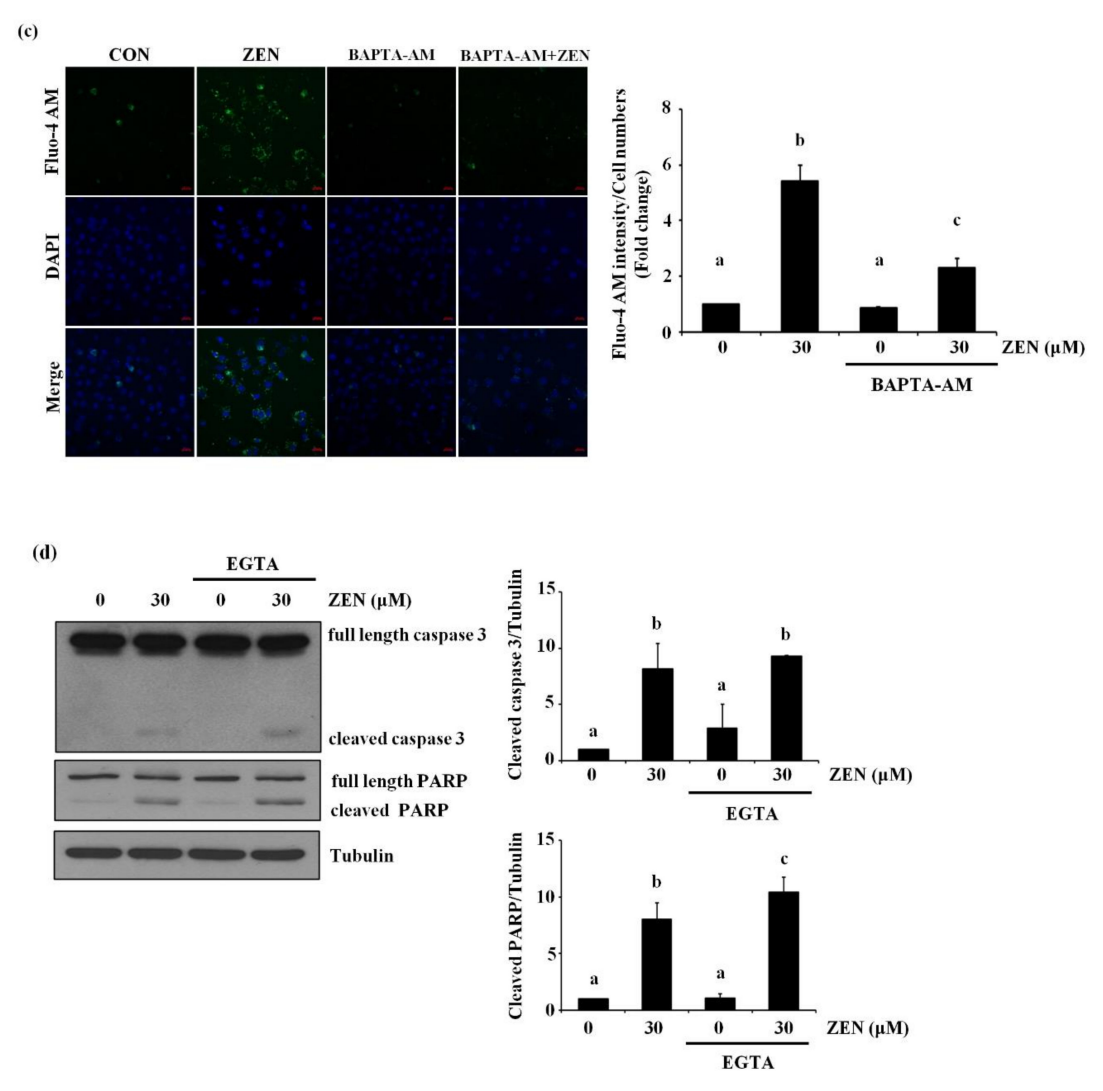
Cytosolic Ca^2+^ is involved in ZEN-induced EC apoptosis. After pretreatment with 2 µM of BAPTA-AM (**a**–**c**) or 0.1 mM of EGTA (**d**) for 1 h, BAECs were incubated with 30 µM of ZEN for 24 h. (**a**) Cell viability was measured using the MTT assay. (**b**,**d**) The protein expression of cleaved caspase 3 and PARP relative to tubulin was quantified using western blot analyses. The plots depict the mean fold changes relative to the control (±SD) from at least four independent experimental trials. (**c**) Visualization of cytosolic Ca^2+^ in the BAECs stained with the membrane-permeable Ca^2+^ indicator dye Fluo-4 AM using a confocal microscope (100×). The different alphabetical letters refer to significant difference (*p* < 0.05) among groups, which were determined by one-way ANOVA followed by Tukey’s multiple comparisons.

It has also been reported that ZEN increases cell death by increasing level of cytosolic Ca^2+^ [23,24]. Therefore, we examined whether ZEN induced EC apoptosis by increasing the level of cytosolic Ca^2+^ in BAECs. Pretreatment with the cytosolic Ca^2+^ chelator BAPTA-AM prior to ZEN exposure prevented ZEN-induced cell death of BAECs, as shown in Figure 7a. Furthermore, caspase 3 and PARP cleavage as well as ERK1/2 and p53 phosphorylation induced by ZEN were also reversed by BAPTA-AM treatment (Figure 7b). As shown in Figure 7c, using confocal microscopy, we confirmed that the ZEN-induced cytosolic Ca^2+^ was reduced by BAPTA-AM. Interestingly, EGTA, an extracellular Ca^2+^ chelator, did not have the same effect as BAPTA-AM on the ZEN-treated BAECs (Figure 7d). From these results, we postulated that releasing cytosolic Ca^2+^ is likely the primary upstream event for triggering ERK1/2/p53/caspase 3-mediated apoptotic pathway by ZEN exposure.

### 2.8. The Endoplasmic Reticulum (ER) Is the Organelle Responsible for Cytosolic Ca^2+^ Release by ZEN

**Figure 8:**
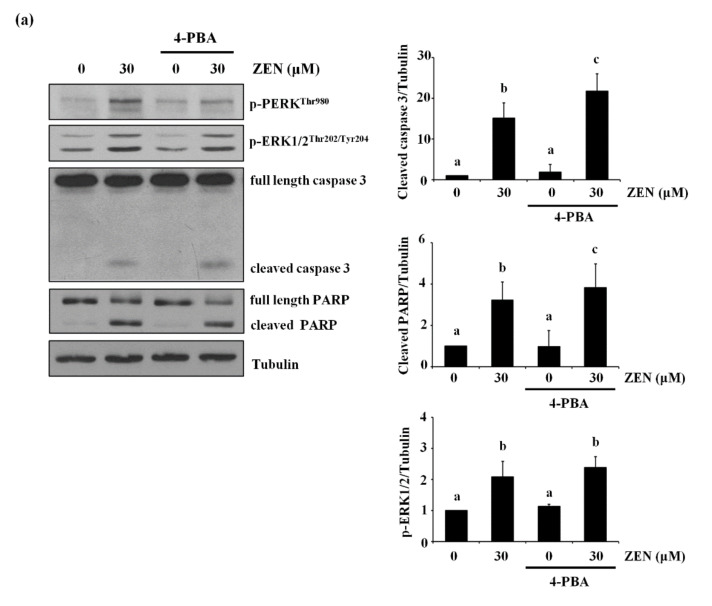

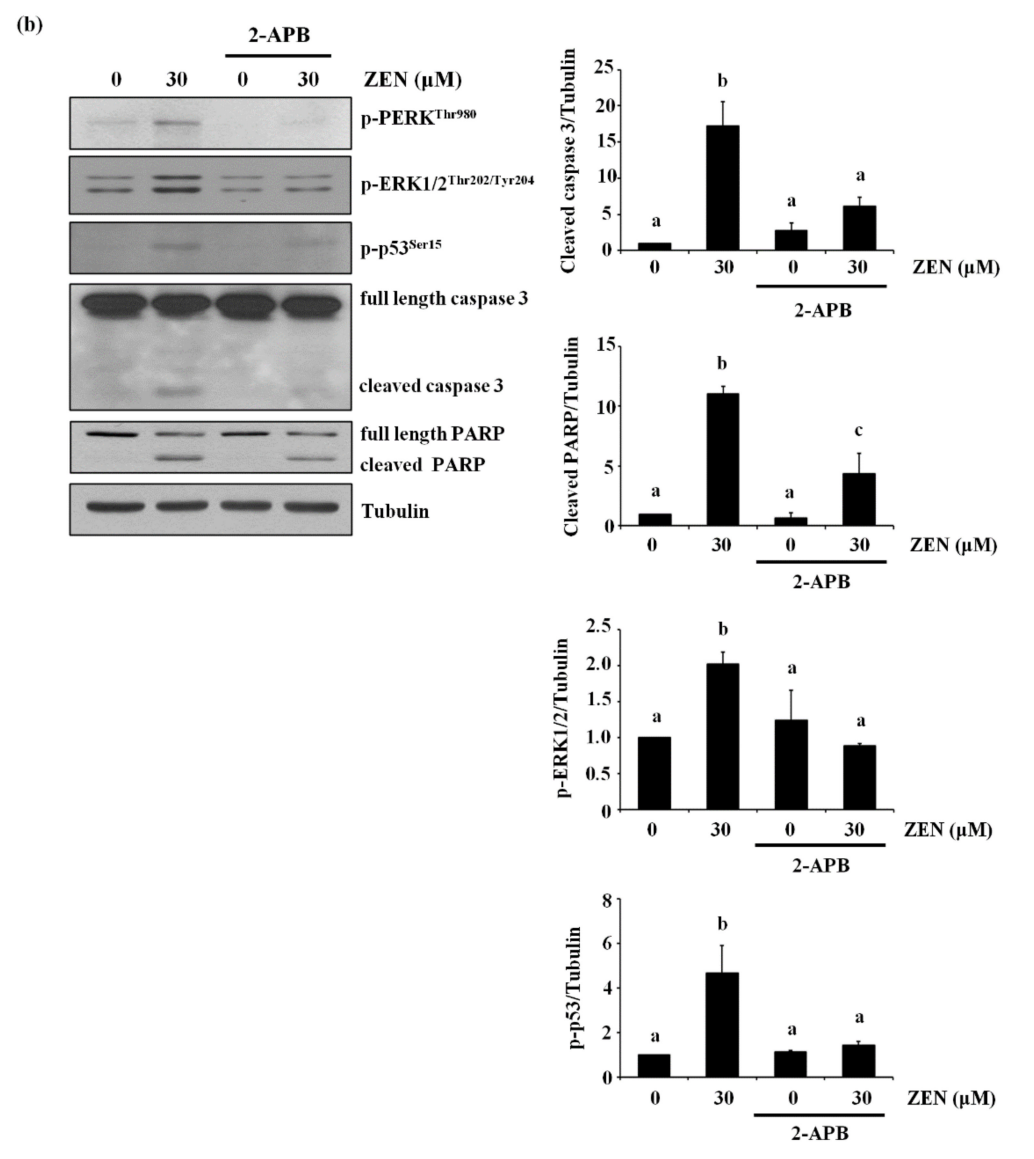
ER-mediated Ca^2+^ channel, but not ER stress, is involved in ZEN-induced apoptosis of BAECs. After pretreatment with (**a**) 2 mM of 4-PBA, an ER stress inhibitor, or (**b**) 20 µM of 2-APB, an ER-mediated Ca^2+^ channel inhibitor, for 1 h, BAECs were incubated with 30 µM of ZEN for 24 h. (**a**,**b**) The protein expression of cleaved caspase 3 and PARP relative to tubulin was quantified using western blot analyses. The plots depict the mean fold changes relative to the control (±SD) from at least four independent experimental trials. The different alphabetical letters refer to significant difference (*p* < 0.05) among groups, which were determined by one-way ANOVA followed by Tukey’s multiple comparisons.

ZEN was reported to increase ER stress to induce apoptosis [12,25,26,27] and the ER is the major organelle responsible for storing and releasing Ca^2+^ [28]. Under our conditions, we examined whether ER stress was involved in the effects of ZEN on BAEC apoptosis. It has been known that when ER stress is induced, the phosphorylation of PERK is increased, which then activates the PERK/eIF2α-dependent pro-apoptotic transcriptional signal to induce apoptosis [29,30]. For this reason, we chose the level of phosphorylation of PERK as a marker of ER stress. As expected, ZEN increased the phosphorylation of PERK, which is decreased by pretreatment with 4-PBA, an ER stress inhibitor (Figure 8a). However, cleavage of caspase 3 and PARP induced by ZEN was not prevented with 4-PBA, indicating that ER stress is unlikely to be involved in the ZEN-induced apoptosis of BAECs. Lastly, we treated the BAECs with 2-APB, an ER Ca^2+^ channel inhibitor, and found that 2-APB significantly prevented the effects of ZEN on EC apoptosis (Figure 8b). Based on these findings, we concluded that ZEN induces BAEC apoptosis by promoting Ca^2+^ release from ER to elevate cytosolic Ca^2+^, which subsequently activates ERK1/2/p53/caspase 3 apoptotic pathway.

## 3. Discussion

Many studies have focused on the toxicological effects of ZEN on the cells/tissues related to reproductive and digestive function [10,31], but there have been only few studies addressing the effects on vascular function. It has been widely reported that ZEN alters the function and viability of various cells/tissues through interactions with the estrogen receptor [5,6], as well as by inducing ROS and ER stress [10,11,25,26]. Nevertheless, we found that ZEN induces apoptosis of BAECs through a mechanism that is independent of the estrogen receptor and ROS, but is closely associated with cytosolic Ca^2+^ levels and subsequent activation of an ERK1/2/p53/caspase 3 signaling axis. This study provides the first evidence of novel toxicological mechanism of ZEN-induced apoptosis using BAECs.

**Figure 9:**
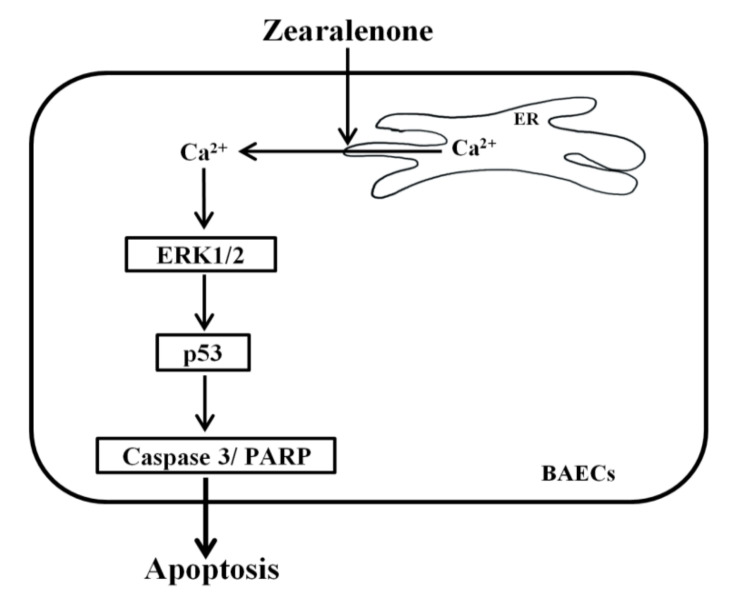
Schematic illustration of the molecular mechanism by which ZEN induces apoptosis in BAECs. ZEN increases the levels of cytosolic Ca^2+^ that are released from ER through ER Ca^2+^ channel activation. The increased cytosolic Ca^2+^ levels stimulate the phosphorylation of ERK1/2 and p53, subsequently enhancing the cleavage of caspase 3 and PARP and resulting in apoptosis of BAECs.

Our study demonstrated that ZEN disrupts vascular function by inducing apoptotic death of ECs, primarily through an ERK1/2/p53/caspase 3 signaling pathway (Figure 9). ZEN was previously shown to induce apoptosis of RAW264.7 cells through a JNK and p38 MAPK pathway [14]. However, we found that ZEN induces apoptosis through an ERK1/2/p53/caspase 3 signaling pathway rather than through JNK and p38 MAPK. Similar to JNK and p38 MAPK, ERK1/2 belongs to the MAPK family, but ERK1/2 is better known as a signaling molecule that promotes cell proliferation. However, its role seems to be largely dependent on drugs and cell types, where it can also induce apoptosis, senescence and autophagy [26]. In this regard, various stimulants, such as DNA damaging agents (etoposide and UV) or anticancer compounds (resveratrol, taxol and oridonin), activate ERK1/2 to induce apoptosis in NIH 3T3, human papillary thyroid carcinoma, human melanoma and human breast adenocarcinoma cell lines [26]. Similar to these compounds, ZEN increases phosphorylation of ERK1/2 to further trigger other downstream pro-apoptotic molecules, including p53 and the caspase cascade of apoptosis, as shown in our study [32,33]. In some cases, p53 can be an activator of ERK1/2, initiating the p53/ERK1/2 signaling pathway [34,35]. However, given that inhibition of ERK1/2 decreased ZEN-stimulated phosphorylation of p53 but not *vice versa*, it is likely that ERK1/2 acts as upstream of p53 to initiate apoptosis of ECs, suggesting that ZEN-induced apoptosis occurs through sequential activation of ERK1/2, p53 and caspase 3.

Several factors are involved in the activation of the ERK1/2/p53/caspase pathway. For example, cytosolic Ca^2+^ release activates molecules such as Ras, G_αi2_, calpain, and calmodulin kinase 1, which then regulate ERK1/2 activity [36,37,38]. In this regard, a previous study reported that calcimycin activates Ras through Ras-guanine nucleotide releasing factor 2, consequently triggering the cytosolic Ca^2+^/ERK1/2/p53 signaling pathway to induce apoptosis of the rabbit lens epithelial cells [39]. Our study also validates the findings of this previous study, where chelation of cytosolic Ca^2+^ using BAPTA-AM, but not extracellular Ca^2+^, effectively prevents apoptosis as well as the expression of pro-apoptotic molecules induced by ZEN. These findings demonstrate an important role for the influx of cytosolic Ca^2+^ in inducing the ERK/1/2/p53/caspase 3-mediated apoptotic signaling pathway in ECs exposed to ZEN. Furthermore, our results also indicate that the ZEN-induced increase in cytosolic Ca^2+^ results from the activation of Ca^2+^ channels located in the ER. These findings are fairly consistent with those of the recent study showing that compound K, a major metabolite of ginsenosides, induces apoptosis by activating ER Ca^2+^ channels known as ryanodine receptors to release ER Ca^2+^ in human lung cancer cells, the A549, and SK-MES-1 cell lines [40]. Although ZEN has also been reported to increase cytosolic Ca^2+^ level by inducing ER stress in TM4 cells [41], ovarian cells from pre-pubertal bitches [42] and lymphocytes of chickens [24], our data do not support the involvement of ER stress in ZEN-induced apoptosis. At present, the reason for these inconsistencies has not been determined, but they may be attributable to the different cell types used in our study; BAECs versus TM4 and ovarian cells.

Many studies have reported that ZEN generates ROS, which in turn induces DNA damage and cell death. For example, induction of ROS and malondialdehyde production, as well as loss of mitochondrial membrane potential by ZEN leads to apoptotic death of SIEC02 and SHSY-5Y cells [10,11]. We also found increased ROS production with ZEN treatment, but complete inhibition of ROS production by NAC had no effect on the apoptosis induced by ZEN, as shown in Figure 6. Consistent with our findings, in a study examining DNA damage as an indicator of apoptosis in human embryonic kidney cell line HEK293 cells, ZEN-induced cell death is found to be mediated through a ROS-independent pathway [8], and blocking of ROS using the antioxidant hydroxytyrosol did not prevent the ZEN-induced DNA damage. The authors concluded that lysosomal injury is a potential cause of DNA damage and apoptosis induced by ZEN. Other ROS inducers, such as artesunate and dihydroartemisinin, are also known to mediate apoptotic cell death through a ROS-independent pathway; the former induces Bax-mediated apoptosis in HepG2 cells [43], whereas the latter induces apoptosis through activation of the p38 MAPK/caspase 9/caspase 3 signaling pathway in leukemia HL-60 cells [44].

ZEN has long been known to be an estrogenic mycotoxin that competitively binds to the estrogen receptors to alter the synthesis and secretion of steroidal reproductive hormones [4] as well as sperm quality of rats by increasing apoptosis and necrosis [5]. Therefore, we examined the potential involvement of genomic and nongenomic estrogen receptors in the ZEN-induced apoptotic death of BAECs. Under our experimental conditions, however, ZEN-mediated apoptosis of BAECs was not prevented by adding the inhibitors of either genomic or nongenomic estrogen receptor, as shown in Figure 5. These findings indicate that estrogenic activity mediated by ZEN is unlikely to induce apoptosis, at least for ECs.

## 4. Conclusions

In conclusion, our study is the first to demonstrate that ZEN induces EC apoptosis by activating a signaling axis of cytosolic Ca^2+^/ERK1/2/p53, as illustrated in Figure 9. This molecular mechanism represents a potential risk of ZEN exposure in relation to vascular health in animals and humans.

## 5. Materials and Methods

### 5.1. Materials

ZEN, ICI 182,780 (genomic estrogen receptor antagonist), N-acetyl-L-cysteine (NAC; antioxidant), sodium phenylbutyrate (4-PBA; endoplasmic reticulum (ER) stress inhibitor), BAPTA-AM (cytosolic calcium chelator), U0126 (ERK inhibitor), pifithrin-α (p53 inhibitor), SP600125 (JNK inhibitor), SB203580 (p38 MAPK inhibitor), 2′,7′-dichlorofluorescin-diacetate (DCF-DA) and ethylene glycol-*bis*(2-aminoethylether)-*N*,*N*,*N*′,*N*′-tetraacetic acid (EGTA; extracellular Ca^2+^ chelator) were purchased from Merck (Darmstadt, Germany). Z-DVED-FMK (caspase 3 inhibitor) and G-15 (nongenomic estrogen receptor antagonist) were purchased Tocris bioscience (Bristol, UK). Antibodies against ERK1/2 and all corresponding secondary antibodies were obtained from Santa Cruz Biotechnology (Dallas, TX, USA). Antibody against α-tubulin was purchased from GW Vitek (Seoul, Korea), Antibodies against caspase 3, poly ADP-ribose polymerase (PARP), phosphorylated p44/42 MAPK (ERK1/2) (p-ERK1/2^Thr202/Tyr204^), p-JNK^Thr183/Tyr15^, JNK, p-MAP kinase-activated protein kinase (MAPKAPK)-Thr334 (p-MAPKAPK^Thr334^), MAPKAPK, p-p53^Ser15^ and p-PERK^Thr980^ were purchased from Cell Signaling Technology (Danvers, MA, USA). Lipofectamine 2000, minimal essential medium (MEM), Dulbecco’s phosphate-buffered saline (DPBS), newborn calf serum (NCS), penicillin-streptomycin antibiotics, L-glutamine, trypsin-EDTA solution and plasticware for cell culture were obtained from Thermo Fisher Scientific (Waltham, MA, USA). All other chemicals were of the purest analytical grade.

### 5.2. Cell Culture and Drug Treatments

BAECs were isolated and cultured as described previously [45] and maintained in MEM supplemented with 5% NCS at 37 °C under 5% CO_2_. Cells between passages 5 and 9 were used in 1% NCS for all experiments. The cells were incubated with ZEN at various concentrations for 24 h or with 30 μM ZEN for 4, 8, 16 or 24 h. In separate experiments, various chemicals were added to the cell cultures 1 or 3 h prior to ZEN treatment.

### 5.3. Cell Viability Assay

The cell viability assay was carried out as described [46] using 3-(4,5-dimethylthiazol-2-yl)-2,5-diphenyltetrazolium bromide (MTT, Merck) with minor modifications. BAECs were seeded at a density of 1.0 × 10^4^ per well in a 96-well culture plate (four replicates for each treatment), and incubated with ZEN at various concentrations (0, 10, 30 or 60 μM) for 24 h or with 30 μM ZEN for various time points (4, 8, 16 or 24 h). After the ZEN treatments, the cells were incubated with 5 mg/mL MTT and further incubated for 1 h at 37 °C. The cells were then treated with dimethylsulfoxide (DMSO) for 10 min, and the absorbance was read at 570 nm using a 96-well microtiter plate reader (BioTek Instruments, Winooski, VT, USA).

### 5.4. Annexin V-Fluorescein Isothiocynate (FITC)/Propidium Iodide (PI) Double Staining

Apoptosis of BAECs was measured using annexin V-FITC/PI apoptosis detection kit (BD Biosciences, Franklin Lakes, NJ, USA). Briefly, cells were trypsinized and harvested after centrifugation at 2000× *g* for 3 min, then re-suspended in 500 μL of binding buffer. Annexin-FITC (5 μL) and PI (5 μL) were to the harvested cells in solution and incubated for 15 min at 37 °C in the dark. Scattering signals were detected by fluorescence-activated single cell sorting (FACS) analysis using a flow cytometer (ACEA Biosciences, San Diego, CA, USA). In this study, the percentage of apoptotic cell population was assessed from the annexin V+/PI− population that represents early apoptotic cells.

### 5.5. Detection of Intracellular ROS Levels

Intracellular ROS was measured using the oxidation-sensitive fluorescent probe DCF-DA (Merck), in an assay based on the ROS-dependent oxidation of DCF-DA to 2′,7′-dichlorofluorescein (DCF), as described previously [47] with minor modifications. Briefly, BAECs grown in 96-well culture plates were incubated with 20 μM DCF-DA for 30 min. The DCF-DA solution was removed and the cells were washed with DPBS. The cells were then incubated with NAC for 3 h before ZEN treatment and incubated for an additional 24 h at 37 °C under 5% CO_2_ after the addition of ZEN. The deposited intracellular DCF-DA was measured using a 96-well microtiter plate reader (ex 485 nm/em 530 nm; BioTek Instrument).

### 5.6. Small Interfering RNA (siRNA) Transfection

BAECs were transfected with 100 nM of small interfering RNA (siRNA) using Dharmafect 4 (Dharmacon, Lafayette, CO, USA) according to the manufacturer’s instructions. siRNA oligonucleotides for ERK1, ERK2, p53 and negative control siRNA were purchased from GenePharma (Shanghai, China) as follows: *ERK1*, CCU UUG AGC AUC AGA CCU ACU, CCA AGG AAC GAC UGA AGG AGC; *ERK2*, AGA AAA UCA GCC CUU UUG AGC, AAA UCA UGU UGA AUU CCA AGG; *p53*, ACU ACA AUU UCA UGU GUA ACA, GGU UUA AAC GCU AUG AGA UGU, CAU ACA CUG GGU UGG AAA ACU; and the negative control (N.C.), UUC UCC GAA CGU GUC ACG UTT. At least two sequences of siRNA oligonucleotides were designed and combined to increase the efficiency of knockdown of the gene of interest. The BAECs with or without transfections were grown to confluence and maintained for an additional 24 h in MEM with 1% NCS containing 30 μM ZEN.

### 5.7. Western Blot Analyses

For the western blot analyses, BAECs treated with ZEN in the absence or presence of various additional chemicals were washed with ice-cold Dulbecco’s phosphate-buffered saline (DPBS) and lysed with lysis buffer (20 mM Tris-HCl at pH 7.5, 150 mM NaCl, 1% Triton X-100, 1 mM EDTA, 1 mM EGTA) containing Protease Inhibitor Cocktail™ (Merck), 1 mM β-glycerophosphate, 1 mM phenylmethanesulfonyl fluoride, 1 mM NaF and 1 mM Na_3_VO_4_. The protein concentrations were determined using a BCA protein assay (Merck). Equal quantities of protein (20 μg) were separated on sodium dodecyl sulfate polyacrylamide gel under reducing conditions and then electrophoretically transferred onto nitrocellulose membranes. The blots were then probed with appropriate antibodies, each at a 1:1000 dilution, followed by the corresponding secondary antibodies, and finally developed using enhanced chemiluminescence reagents (ECL, GE Healthcare, Chicago, IL, USA). Proteins on the nitrocellulose membranes were quantified using Image J software (National Institutes of Health, Bethesda, MD, USA). The tubulin was used as a loading control to normalize the quantified values of target proteins of interest.

### 5.8. Reverse Transcription-Polymerase Chain Reaction (PCR)

Total RNA was extracted from BAECs using TRIzol™ Reagent (Thermo Fisher Scientific) as described previously [48]. Briefly, the cells were homogenized in 1 mL of TRIzol™ reagent. The total RNA was then converted to cDNA using SuperScript™ III reverse transcriptase (Thermo Fisher Scientific). PCR amplification of a cDNA encoding each target gene was conducted using the following primers: *p53*-F, 5′-CTA CCA ACA CCA GCT CCT-3′; *p53*-R, 5′-CGG CTC ACA GTA AAA ACC TT-3′; *GAPDH*-F, 5′-TCA CCA GGG CTG CTT TTA AT-3′; *GAPDH*-R, 5′-GGT CAT AAG TCC CTC CAC GA-3′. The amplified products were separated using a 1% agarose gel in TAE buffer (40 mM Tris-acetate, pH 8.0, 1 mM EDTA). The band of the target gene was visualized under UV light using RedSafe™ Nucleic acid Staining Solution (iNtRON biotechnology, Gyeonggi-do, Korea).

### 5.9. Measurement of Cytosolic Ca^2+^ Levels

Cytosolic Ca^2+^ levels were measured using the membrane-permeable Ca^2+^ indicator dye Fluo-4 AM (Thermo Fisher Scientific), as described previously [49]. BAECs were pretreated with BAPTA-AM for 1 h before ZEN treatment in the presence of 1 μM Fluo-4 AM. Fluorescent images were obtained using a confocal microscope (LSM5 Pascall, Carl ZEISS, Oberkochen, Germany). The cytosolic Ca^2+^ level was quantified by measuring the fluorescence intensity (Green) using Image J software, and the number of cells from each image was estimated by counting the nucleus stained with DAPI (Blue). The fluorescence intensity of cytosolic Ca^2+^ level was then normalized by dividing the counted cell numbers.

### 5.10. Statistical Analyses

All statistical analyses were performed using GraphPad Prism software ver. 5 (GraphPad Software, San Diego, CA). Data are expressed as the mean ± standard deviation (SD) and statistical significance (*p* < 0.05) was determined using one-way ANOVA followed by Tukey’s multiple comparison test. The significant differences are denoted with different alphabetical letters.

## Data Availability

Not applicable.

## Supplementary Materials

The following are available online at https://www.mdpi.com/2072-6651/13/3/187/s1. Figure S1. The inhibitory effect of ICI182,780 on eNOS mRNA expression in BAECs. After pretreatment with 10 μM of ICI182,780 for 1 h, BAECs were incubated with 1 nM 17β -estradiol for 24 h. The eNOS mRNA expression was quanti-fied using qRT-PCR. The plots depict the mean fold changes relative to control (±SD) from at least three independent experimental trials. The different alpha-betical letters refer to significant differences (*p* < 0.05) among groups, which were determined by one-way ANOVA followed by Tukey’s multiple comparison, Figure S2. The inhibitory effect of G-15 on the expression of total eNOS and p-eNOSSer1179 in BAECs. After pretreatment with 1 μM of G-15 for 1 h, BAECs were incubated with 100 nM G-1 for 24 h. The protein expression of p-eNOSSer1179 relative to eNOS was quantified using Western blot analyses. The plots depict the mean fold changes relative to control (±SD) from at least three independent experimental trials. The different alphabetical letters refer to significant differ-ences (*p* < 0.05) among groups, which were determined by one-way ANOVA followed by Tukey’s multiple comparison.

## References

[1] Hossain Z., Mari N., Goto T. (2015). The relationship between ergosterol and mycotoxin contamination in maize from various countries. Mycotoxin Res..

[2] Belhassen H., Jimenez-Diaz I., Ghali R., Ghorbel H., Molina-Molina J.M., Olea N., Hedili A. (2014). Validation of a UHPLC-MS/MS method for quantification of zearalenone, alpha-zearalenol, beta-zearalenol, alpha-zearalanol, be-ta-zearalanol and zearalanone in human urine. J. Chromatogr. B.

[3] Rogowska A., Pomastowski P., Sagandykova G., Buszewski B. (2019). Zearalenone and its metabolites: Effect on human health, metabolism and neutralisation methods. Toxicon.

[4] Zheng W., Feng N., Wang Y., Noll L., Xu S., Liu X., Lu N., Zou H., Gu J., Yuan Y. (2019). Effects of zearalenone and its derivatives on the synthesis and secretion of mammalian sex steroid hormones: A review. Food Chem. Toxicol..

[5] Adibnia E., Razi M., Malekinejad H. (2016). Zearalenone and 17 beta-estradiol induced damages in male rats reproduction potential; evidence for ERalpha and ERbeta receptors expression and steroidogenesis. Toxicon.

[6] He J., Wei C., Li Y., Liu Y., Wang Y., Pan J., Liu J., Wu Y., Cui S. (2018). Zearalenone and alpha-zearalenol inhibit the synthesis and secretion of pig follicle stimulating hormone via the non-classical estrogen membrane receptor GPR30. Mol. Cell. Endocrinol..

[7] Zatecka E., Ded L., Elzeinova F., Kubátová A., Dorosh A., Margaryan H., Dostalova P., Korenkova V., Hošková K., Pěknicová J. (2014). Effect of zearalenone on reproductive parameters and expression of selected testicular genes in mice. Reprod. Toxicol..

[8] Gao F., Jiang L.-P., Chen M., Geng C.-Y., Yang G., Ji F., Zhong L.-F., Liu X.-F. (2013). Genotoxic effects induced by zearalenone in a human embryonic kidney cell line. Mutat. Res. Toxicol. Environ. Mutagen..

[9] Lu J., Yu J.Y., Lim S.S., Son Y.O., Kim D.H., Lee S.A., Shi X., Lee J.C. (2013). Cellular mechanisms of the cytotoxic effects of the zearalenone metabolites alpha-zearalenol and beta-zearalenol on RAW264 7 macrophages. Toxicol. In Vitro.

[10] Zheng W.-L., Wang B.-J., Wang L., Shan Y.-P., Zou H., Song R.-L., Wang T., Gu J.-H., Yuan Y., Liu X.-Z. (2018). ROS-Mediated Cell Cycle Arrest and Apoptosis Induced by Zearalenone in Mouse Sertoli Cells via ER Stress and the ATP/AMPK Pathway. Toxins.

[11] Venkataramana M., Nayaka S.C., Anand T., Rajesh R., Aiyaz M., Divakara S., Murali H., Prakash H., Rao P.L. (2014). Zearalenone induced toxicity in SHSY-5Y cells: The role of oxidative stress evidenced by N-acetyl cysteine. Food Chem. Toxicol..

[12] Lin P., Chen F., Sun J., Zhou J., Wang X., Wang N., Li X., Zhang Z., Wang A., Jin Y. (2015). Mycotoxin zearalenone induces apoptosis in mouse Leydig cells via an endoplasmic reticulum stress-dependent signalling pathway. Reprod. Toxicol..

[13] Wang Y., Zheng W., Bian X., Yuan Y., Gu J., Liu X., Liu Z., Bian J. (2014). Zearalenone induces apoptosis and cytoprotective autophagy in primary Leydig cells. Toxicol. Lett..

[14] Yu J.-Y., Zheng Z.-H., Son Y.-O., Shi X., Jang Y.-O., Lee J.-C. (2011). Mycotoxin zearalenone induces AIF- and ROS-mediated cell death through p53- and MAPK-dependent signaling pathways in RAW264.7 macrophages. Toxicol. Vitr..

[15] Anderson C., Majeste A., Hanus J., Wang S. (2016). E-Cigarette Aerosol Exposure Induces Reactive Oxygen Species, DNA Damage, and Cell Death in Vascular Endothelial Cells. Toxicol. Sci..

[16] Ouyang J., Li R., Shi H., Zhong J. (2019). Curcumin Protects Human Umbilical Vein Endothelial Cells against H_2_O_2_-Induced Cell Injury. Pain Res. Manag..

[17] Ahmad S., Alam Khan S., Kindelin A., Mohseni T., Bhatia K., Hoda N., Ducruet A.F. (2019). Acetyl-11-keto-β-boswellic acid (AKBA) Attenuates Oxidative Stress, Inflammation, Complement Activation and Cell Death in Brain Endothelial Cells Following OGD/Reperfusion. Neuro Mol. Med..

[18] Gajecki M. (2002). Zearalenone—Undesirable substances in feed. Pol. J. Veter-Sci..

[19] Zheng W., Wang B., Li X., Wang T., Zou H., Gu J., Yuan Y., Liu X., Bai J., Bian J. (2018). Zearalenone Promotes Cell Proliferation or Causes Cell Death?. Toxins.

[20] Wang H., Zhao X., Ni C., Dai Y., Guo Y. (2018). Zearalenone regulates endometrial stromal cell apoptosis and migration via the promotion of mitochondrial fission by activation of the JNK/Drp1 pathway. Mol. Med. Rep..

[21] Pistol G.C., Braicu C., Motiu M., Gras M.A., Marin D.E., Stancu M., Calin L., Israel-Roming F., Berindan-Neagoe I., Taranu I. (2015). Zearalenone mycotoxin affects immune mediators, MAPK signalling molecules, nuclear receptors and ge-nome-wide gene expression in pig spleen. PLoS ONE.

[22] Ayed-Boussema I., Bouaziz C., Rjiba K., Valenti K., Laporte F., Bacha H., Hassen W. (2008). The mycotoxin Zearalenone induces apoptosis in human hepatocytes (HepG2) via p53-dependent mitochondrial signaling pathway. Toxicol. Vitr..

[23] Jilani K., Lang F. (2013). Ca2+-dependent suicidal erythrocyte death following zearalenone exposure. Arch. Toxicol..

[24] Wang Y.C., Deng J.L., Xu S.W., Peng X., Zuo Z.C., Cui H.M., Ren Z.H., Wang Y. (2012). Effects of zearalenone on calcium homeostasis of splenic lymphocytes of chickens in vitro. Poult. Sci..

[25] Wang M., Kern A.M., Hülskötter M., Greninger P., Singh A., Pan Y., Chowdhury D., Krause M., Baumann M., Benes C.H. (2014). EGFR-Mediated Chromatin Condensation Protects KRAS-Mutant Cancer Cells against Ionizing Radiation. Cancer Res..

[26] Cagnol S., Chambard J.C. (2010). ERK and cell death: Mechanisms of ERK-induced cell death—Apoptosis, autophagy and se-nescence. FEBS J..

[27] Chen F., Li Q., Zhang Z., Lin P., Lei L., Wang A., Jin Y. (2015). Endoplasmic Reticulum Stress Cooperates in Zeara-lenone-Induced Cell Death of RAW 264.7 Macrophages. Int. J. Mol. Sci..

[28] Celli A., Crumrine D., Meyer J.M., Mauro T.M. (2016). Endoplasmic Reticulum Calcium Regulates Epidermal Barrier Response and Desmosomal Structure. J. Investig. Dermatol..

[29] Sano R., Reed J.C. (2013). ER stress-induced cell death mechanisms. Biochim. Biophys. Acta (BBA) Bioenerg..

[30] Romine I.C., Wiseman R.L. (2019). PERK Signaling Regulates Extracellular Proteostasis of an Amyloidogenic Protein During Endoplasmic Reticulum Stress. Sci. Rep..

[31] Zhu L., Yuan H., Guo C., Lu Y., Deng S., Yang Y., Wei Q., Wen L., He Z. (2012). Zearalenone induces apoptosis and necrosis in porcine granulosa cells via a caspase-3- and caspase-9-dependent mitochondrial signaling pathway. J. Cell. Physiol..

[32] Wang Y., Xu H., Lu Z., Yu X., Lv C., Tian Y., Sui D. (2018). Pseudo-Ginsenoside Rh2 induces A549 cells apoptosis via the Ras/Raf/ERK/p53 pathway. Exp. Ther. Med..

[33] Yang S.-H., Wang S.-M., Syu J.-P., Chen Y., Wang S.-D., Peng Y.-S., Kuo M.-F., Kung H.-N. (2014). Andrographolide Induces Apoptosis of C6 Glioma Cells via the ERK-p53-Caspase 7-PARP Pathway. BioMed Res. Int..

[34] Lee S.Y., Shin S.J., Kim H.S. (2013). ERK1/2 activation mediated by the nutlin3induced mitochondrial translocation of p53. Int. J. Oncol..

[35] Lee S.Y., Choi H.C., Choe Y.J., Shin S.J., Lee S.H., Kim H.S. (2014). Nutlin-3 induces BCL2A1 expression by activating ELK1 through the mitochondrial p53-ROS-ERK1/2 pathway. Int. J. Oncol..

[36] Holstein D.M., Berg K.A., Leeb-Lundberg L.M., Olson M.S., Saunders C. (2004). Calcium-sensing receptor-mediated ERK1/2 activation requires Galphai2 coupling and dynamin-independent receptor internalization. J. Biol. Chem..

[37] Veeranna, Kaji T., Boland B., Odrljin T., Mohan P., Basavarajappa B.S., Peterhoff C., Cataldo A., Rudnicki A., Amin N. (2004). Calpain mediates calcium-induced activation of the erk1,2 MAPK pathway and cytoskeletal phosphorylation in neurons: Relevance to Alzheimer’s disease. Am. J. Pathol..

[38] Schmitt J.M., Wayman G.A., Nozaki N., Soderling T.R. (2004). Calcium Activation of ERK Mediated by Calmodulin Kinase I. J. Biol. Chem..

[39] Li D.W.-C., Liu J.-P., Mao Y.-W., Xiang H., Wang J., Ma W.-Y., Dong Z., Pike H.M., Brown R.E., Reed J.C. (2005). Calcium-activated RAF/MEK/ERK Signaling Pathway Mediates p53-dependent Apoptosis and Is Abrogated by αB-Crystallin through Inhibition of RAS Activation. Mol. Biol. Cell.

[40] Shin D.-H., Leem D.-G., Shin J.-S., Kim J.-I., Kim K.-T., Choi S.Y., Lee M.-H., Choi J.-H., Lee K.-T. (2018). Compound K induced apoptosis via endoplasmic reticulum Ca2+ release through ryanodine receptor in human lung cancer cells. J. Ginseng Res..

[41] Feng N., Wang B., Cai P., Zheng W., Zou H., Gu J., Yuan Y., Liu X., Liu Z., Bian J. (2020). ZEA-induced autophagy in TM4 cells was mediated by the release of Ca(2+) activates CaMKKbeta-AMPK signaling pathway in the endoplasmic reticulum. Toxicol. Lett..

[42] Gajęcka M. (2012). The effect of low-dose experimental zearalenone intoxication on the immunoexpression of estrogen receptors in the ovaries of pre-pubertal bitches. Pol. J. Veter-Sci..

[43] Qin G., Wu L., Liu H., Pang Y., Zhao C., Wu S., Wang X., Chen T. (2015). Artesunate induces apoptosis via a ROS-independent and Bax-mediated intrinsic pathway in HepG2 cells. Exp. Cell Res..

[44] Lu J.J., Meng L.H., Cai Y.J., Chen Q., Tong L.J., Lin L.P., Ding J. (2008). Dihydroartemisinin induces apoptosis in HL-60 leu-kemia cells dependent of iron and p38 mitogen-activated protein kinase activation but independent of reactive oxygen species. Cancer Biol. Ther..

[45] Kim H.P., Lee J.Y., Jeong J.K., Bae S.W., Lee H.K., Jo I. (1999). Nongenomic Stimulation of Nitric Oxide Release by Estrogen Is Mediated by Estrogen Receptor α Localized in Caveolae. Biochem. Biophys. Res. Commun..

[46] Kim J.-Y., Choi J.-Y., Lee H.-J., Byun C.J., Park J.-H., Park J.H., Cho H.-S., Cho S.-J., Jo S.A., Jo I. (2015). The Green Tea Component (-)-Epigallocatechin-3-Gallate Sensitizes Primary Endothelial Cells to Arsenite-Induced Apoptosis by Decreasing c-Jun N-Terminal Kinase-Mediated Catalase Activity. PLoS ONE.

[47] Tsai M.H., Liu J.F., Chiang Y.C., Hu S.C., Hsu L.F., Lin Y.C., Lin Z.C., Lee H.C., Chen M.C., Huang C.L. (2017). Arto-carpin, an isoprenyl flavonoid, induces p53-dependent or independent apoptosis via ROS-mediated MAPKs and Akt ac-tivation in non-small cell lung cancer cells. Oncotarget.

[48] Cho D.H., Choi Y.J., Jo S.A., Jo I. (2004). Nitric oxide production and regulation of endothelial nitric-oxide synthase phos-phorylation by prolonged treatment with troglitazone: Evidence for involvement of peroxisome proliferator-activated receptor (PPAR) gamma-dependent and PPARgamma-independent signaling pathways. J. Biol. Chem..

[49] Kim H.Y., Yu Y., Oh S.-Y., Wang K.-K., Kim Y.-R., Jung S.-C., Kim H.S., Jo I. (2019). Far-Infrared Irradiation Inhibits Adipogenic Differentiation and Stimulates Osteogenic Differentiation of Human Tonsil-Derived Mesenchymal Stem Cells: Role of Protein Phosphatase 2B. Cell. Physiol. Biochem..

