# Supplementary material for: Zearalenone Induces Endothelial Cell Apoptosis through Activation of a Cytosolic Ca2+/ERK1/2/p53/Caspase 3 Signaling Pathway
Source: Toxins (Basel). 2021 Mar 4;13(3):187. doi: 10.3390/toxins13030187 (PMC8001463; doi:10.3390/toxins13030187)
Supplement: Supplementary file 1 [file toxins-13-00187-s001.pdf]

Supplementary Materials: Zearalenone Induces Endothelial Cell Apoptosis through Activation of a Cytosolic Ca^{2+}/ERK1/2/p53/Caspase 3 Signaling Pathway

Hyeon-Ju Lee, Se-Young Oh and Inho Jo

1. Methods

1.1. Quantitative real-time polymerase chain reaction (qRT-PCR)

Total RNA was isolated using a Trizol™ Reagent as described previously [1]. Briefly, the cells were homogenized in 1 mL of TRIzol™ reagent. The total RNA was then converted to cDNA using SuperScript™ III reverse transcriptase. PCR amplification of cDNA encoding each target gene was conducted using the following primers: *eNOS*-F, 5′-GAG TTA CAA GAT CCG CTT CA-3′ and *eNOS*-R, 5′-AGT CCG AAC ACA CAG AAC CT-3′; *GAPDH*-F, 5′-ACG TGT CTG TTG TGG ATC TG-3′ and *GAPDH*-R, 5′-GTA GCC TAG AAT GCC CTT GA-3′. PCR was performed with Power SYBR™ Green Master Mix (Applied Biosystems, Foster City, CA) using QuantStudio™ 3 Real-Time PCR system (Applied Biosystems). The expression of eNOS relative to GAPDH was quantified using the ΔΔCt method.

1.2. Western blot analyses

For the Western blot analyses, BAECs treated with a nongenomic estrogen receptor agonist G-1 (Tocris bioscience) in the absence or presence of G-15, a nongenomic estrogen receptor antagonist, were washed with ice-cold DPBS and lysed with lysis buffer (20 mM Tris-HCl at pH 7.5, 150 mM NaCl, 1% Triton X-100, 1 mM EDTA, 1 mM EGTA) containing Protease Inhibitor Cocktail™, 1 mM β-glycerophosphate, 1 mM phenylmethanesulfonyl fluoride, 1 mM NaF, and 1 mM Na_3VO_4. The protein concentrations were determined using a BCA protein assay. Equal quantities of protein (20 μg) were separated on sodium dodecyl sulfate polyacrylamide gel under reducing conditions and then electrophoretically transferred onto nitrocellulose membranes. The blots were then probed with phosphorylated eNOS (p-eNOS^{Ser1179}) or eNOS (BD bioscience, Franklin Lakes, NJ) at a 1:1000 dilution or tubulin at a 1:3000, followed by their corresponding secondary antibodies. The membranes were then developed using ECL reagents. Proteins on the nitrocellulose membranes were quantified using Image J software. The tubulin was used as a loading control to normalize the quantified values of target proteins of interest.

2. Results

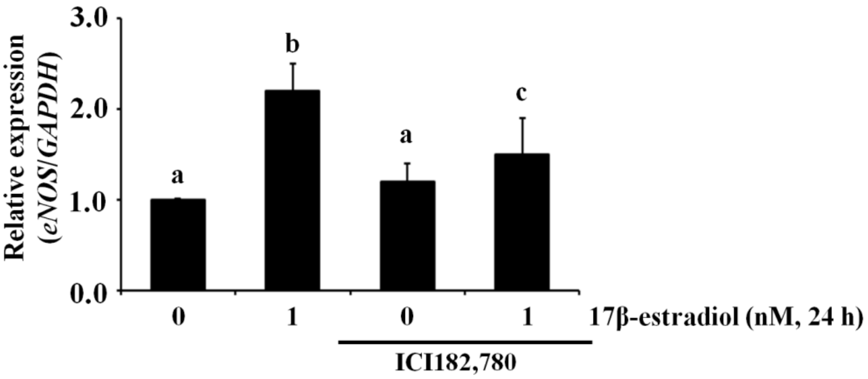

Figure S1. The inhibitory effect of ICI182,780 on eNOS mRNA expression in BAECs. After pretreatment with 10 μM of ICI182,780 for 1 h, BAECs were incubated with 1 nM 17β-estradiol for 24 h. The eNOS mRNA expression was quantified using qRT-PCR. The plots depict the mean fold changes relative to control (± SD) from at least three independent experimental trials. The different alphabetical letters refer to significant differences ($p < 0.05$) among groups, which were determined by one-way ANOVA followed by Tukey's multiple comparisons.

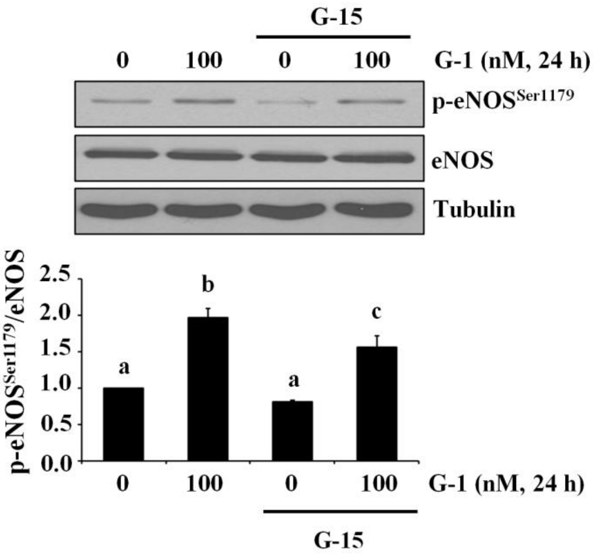

Figure S2. The inhibitory effect of G-15 on the expression of total eNOS and p-eNOS^{Ser1179} in BAECs. After pretreatment with 1 μM of G-15 for 1 h, BAECs were incubated with 100 nM G-1 for 24 h. The protein expression of p-eNOS^{Ser1179} relative to eNOS was quantified using western blot analyses. The plots depict the mean fold changes relative to control (± SD) from at least three independent experimental trials. The different alphabetical letters refer to significant differences ($p < 0.05$) among groups, which were determined by one-way ANOVA followed by Tukey's multiple comparisons.

Reference

1. Cho, D.H.; Choi, Y.J.; Jo, S.A.; Jo, I. Nitric oxide production and regulation of endothelial nitric-oxide synthase phosphorylation by prolonged treatment with troglitazone: evidence for involvement of peroxisome proliferator-activated receptor (PPAR) gamma-dependent and PPARgamma-independent signaling pathways. *J Biol Chem* **2004**, *279*, 2499-2506.
